# Effects of transcranial direct current stimulation on gait in people with Parkinson’s disease: study protocol for a randomized, controlled clinical trial

**DOI:** 10.1186/s13063-018-2982-z

**Published:** 2018-11-29

**Authors:** Vida Alizad, Marcus Meinzer, Laurent Frossard, Remco Polman, Simon Smith, Graham Kerr

**Affiliations:** 1 Movement Neuroscience Program, Institute of Health and Biomedical Innovation, Queensland University of Technology, Brisbane, QLD Australia; 2 Iranian Research Centre on Ageing, The University of Social Welfare and rehabilitation Sciences, Tehran, Iran; 3 Centre for Clinical Research, The University of Queensland, Brisbane, QLD Australia; 4 Institute of Health and Biomedical Innovation Queensland University of Technology, Brisbane, QLD Australia; 5 School of Exercise and Nutrition Sciences, Queensland University of Technology, Brisbane, QLD Australia; 6 Institute for Social Science Research (ISSR), The University of Queensland, Brisbane, QLD Australia

**Keywords:** Parkinson’s disease, Transcranial direct current stimulation, Gait

## Abstract

**Background:**

Gait difficulties are common and frequently devastating to people with Parkinson’s disease (PD). These difficulties are often followed by an increased risk of falls, leading to injury, hospitalization and mortality. The dysfunction in the basal ganglia-thalamocortical motor circuits and reduced activity in the premotor and primary motor cortices has raised interest in transcranial direct current stimulation (tDCS) as an adjunct intervention in PD. tDCS might provide a potentially safe and non-invasive treatment by modulating cortical excitability and behavioural outcomes. The aim of this study is to compare the effects of different monopolar and bipolar montages of tDCS administered to the motor cortex and cerebellum on gait speed in PD.

**Methods:**

This study will be conducted in a randomized, double-blind cross-over design. Eighteen participants diagnosed with Parkinson’s disease will receive anodal and sham tDCS (1 mA, 20 min, 10 × 4 cm^2^) over the premotor and primary motor cortices with the cathode over the cerebellum during treadmill walking. Three montages will be applied over three sessions and compared: anodal tDCS with a small active cathode (4 × 4 cm^2^); anodal tDCS with a large, functionally inert cathode (10 × 10 cm^2^); and sham tDCS. The primary outcome measure is gait speed, and secondary outcome measures include gait parameters (temporospatial, segmental, kinematic), the Timed Up and Go test and lower limb muscle activity patterns as measured by electromyography.

**Discussion:**

This study will investigate the short-term effects of anodal tDCS over the premotor and primary motor cortices on gait abilities using monopolar and bipolar montages in people with PD. The outcomes will inform future studies aimed at inducing longer-lasting changes in neural excitability and performance using multisession tDCS designs in PD.

**Trial registration:**

Australian New Zealand Clinical Trials Registry (ANZCTR), ACTRN12618000063213. Registered on 17 January 2018. Retrospectively registered.

**Electronic supplementary material:**

The online version of this article (10.1186/s13063-018-2982-z) contains supplementary material, which is available to authorized users.

## Background

Gait difficulties are common and often incapacitating for individuals with Parkinson’s disease (PD). They manifest as reduced gait speed, stride length, coordination and arm swing [1] as well as increased cadence, upper body movement and double support duration [2, 3]. Underpinning this altered gait are changes in muscle activation. This includes increased activation of hamstrings and quadriceps in mid-stance and late stance phases and decreased activation of tibialis anterior and gastrocnemius lateralis muscles in early stance phase, which results in delayed heel-off phase and gait initiation [4, 5]. Importantly, gait deficiencies result in a nine-times greater risk of falls [6], leading to a five-times greater risk of sustaining fall-related injuries, hospitalization and mortality for people with PD [3].

Motor complications in PD have been linked to degeneration of dopaminergic neurons in the substantia nigra compacta of the basal ganglia, which results in a deficit of dopamine and disruption of dopaminergic neurotransmission [7]. There is a consequent dysfunction in the basal ganglia-thalamocortical motor circuits with an overactivity of inhibitory efferents from the basal ganglia to the thalamus, resulting in suppression on thalamocortical projections, with abnormal activity in the premotor and primary motor cortices, resulting in gait difficulties [8, 9]. The dopamine deficit and dysfunction in the basal ganglia-thalamocortical motor circuits also affect cortico-cerebellar circuits as they are functionally connected [10, 11]. The cerebellum has been shown to be hyperactive in PD, presumably to compensate for the deficiency in function of the basal ganglia and the cortico-cerebellar circuit [10].

Non-invasive brain stimulation of the premotor and primary motor cortices may modulate neural activity in these neural circuits [10, 12, 13] and re-equilibrate the cortico-cerebellar circuits [14], thereby improving gait ability and lower limb muscle activity in people with PD.

Transcranial direct current stimulation (tDCS) is a non-invasive brain stimulation technique now widely used in neuroscience and clinical research in humans [15, 16]. It has an excellent safety profile [17] and is a low-cost technique suited for double-blind clinical trials [18]. The tDCS process modulates cortical excitability via a weak direct current that is delivered by two or more scalp-affixed electrodes [19]. Anodal tDCS (a-tDCS) typically increases cortical excitability and cathodal tDCS (c-tDCS) decreases it [13]. The underlying mechanisms by which tDCS modulates neurophysiology and behaviour are yet to be fully understood [20]. However, acute effects of a single session (e.g. 20–30 min) might result in transient modulation of the neural resting membrane potential [19, 21]. Further, the cumulative effects of repeated tDCS sessions can be explained by modulation of post-synaptic connections, similar to long-term potentiation and long-term depression, which play a key role in neuroplasticity underlying adaptive human behaviour and learning [19].

Emerging research suggests the potential for a-tDCS over the primary motor cortex (M1) to improve gait difficulties in PD due to the dense connectivity of the cortex and the basal ganglia, and the possibility of targeting the basal ganglia-thalamocortical motor circuits [22–27]. However, results from previous studies have indicated that the effects of tDCS on gait speed [24, 26–34] and stride length [24, 27, 32] have been variable and appear to be dependent on stimulation site and electrode size. In one study, bilateral a-tDCS (3.5 × 5 cm^2^) of either premotor and motor or prefrontal cortices with the cathode (5 × 5 cm^2^) over the mastoid demonstrated significant improvement in gait speed [28]. The most substantial gait improvement was reported by Kaski et al. (2014) after applying bilateral a-tDCS combined with physical training or tango [33, 35]. This bilateral arrangement was employed because gait control is the result of parallel and bilateral involvement of motor and premotor cortices; the authors used a bilateral stimulation set-up, and the anode (10 × 4 cm^2^) was centred over the premotor and primary motor cortices. The cathode (4 × 4 cm^2^) was attached over the inion to ensure current flow through the sensorimotor strip but without affecting the cerebellum. While this montage showed beneficial effects on gait speed and stride length, it remained unclear whether these effects were mediated by sensorimotor effects of the anode, possible inhibitory effects on the cerebellum due to the proximity of the cathode to this region or synergetic effects induced by the bipolar set-up. Therefore, a systematic investigation of electrode size and placement, which most likely determine the extent of tDCS effects on gait abilities in PD, is required.

In the present study, we will address this issue by systematically investigating the effects of a-tDCS over the premotor and primary motor cortices and the synergetic effects of a-tDCS over the premotor and primary motor cortices and c-tDCS over the cerebellum. As it is thought that tDCS is most effective when combined with a behavioural task [36], tDCS will be administered during simultaneous treadmill walking, because a recent study reported more pronounced effects after this task compared to other physical tasks in PD [37]. This ensures that the neural circuits, and particularly M1, are active during application of the tDCS. In this study, we also took advantage of the same anode electrode parameters used in studies by Kaski et al. [30, 33, 35, 38]. Applying bilateral a-tDCS over the premotor and primary motor cortices and comparing the effects of both active (small) and functionally inert (large) cathodes over the cerebellum in PD could disentangle the location of possible tDCS effects on gait abilities in PD. We hypothesize that a large cathode will render stimulation over the cerebellum inactive (i.e. a monopolar M1 set-up), whereas a smaller cathode will exert additional inhibitory effects on the cerebellum (i.e. a bipolar M1 cerebellar set-up) [39]. To our knowledge, no study to date has analysed the gait parameters and electromyography (EMG) of the lower limb focusing specifically on different bilateral tDCS montages.

Thus, this randomized placebo (“sham tDCS)” controlled study aims to examine the short-term effects of (1) monopolar bilateral a-tDCS over the premotor and primary motor cortices (exploratory aim 1); (2) bipolar bilateral a-tDCS over the premotor and primary motor cortices and cerebellum cortex (exploratory aim 2) while walking on a treadmill on gait speed as the main outcome measure and other gait kinematics and EMG in people with PD when optimally medicated. The findings will be reported and disseminated through peer-reviewed journal publication and conference presentations. The results of the study will also be presented for PD support groups who contributed in participant recruitment.

## Methods

### Study design and outline

**Fig. 1:**
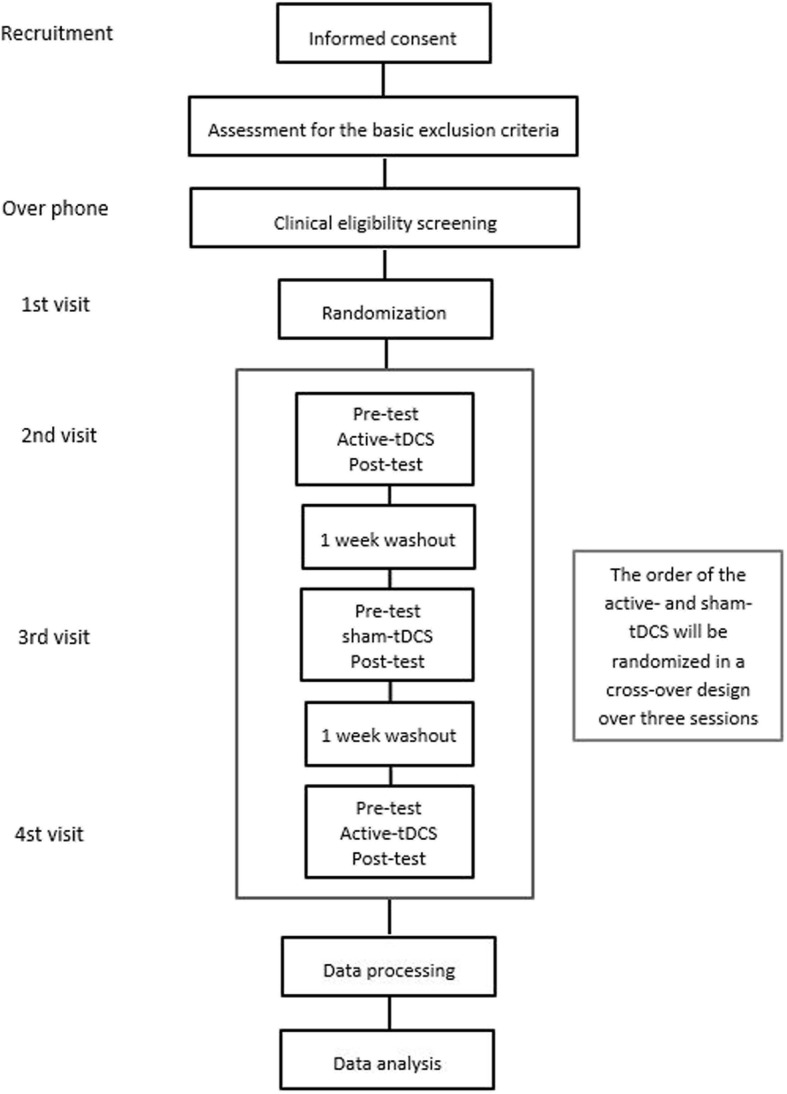
Flowchart of overall study design. Note: Active tDCS is either monopolar bilateral a-tDCS over premotor cortex and primary motor cortex or bipolar bilateral a-tDCS over premotor cortex, primary motor cortex and cerebellum cortex

**Fig. 2:**
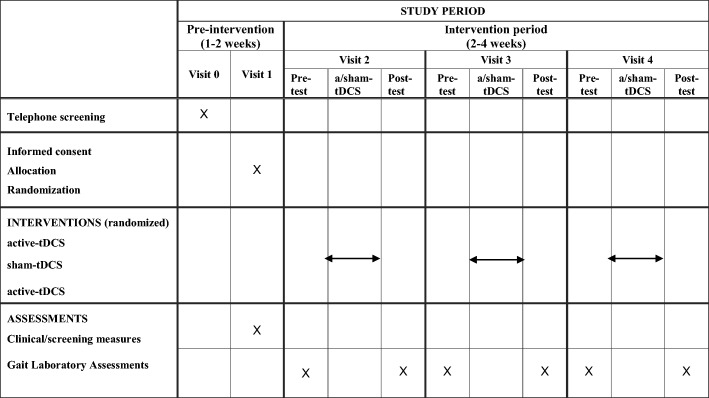
SPIRIT schedule

The study will employ a randomized, double-blind, sham-controlled cross-over design to assess the effect of a-tDCS delivered during treadmill walking on natural overground gait performance and associated muscle activation. Participants will receive bilateral active or sham tDCS for 20 min while walking on a treadmill over three sessions. Gait will be assessed using a three-dimensional (3D) motion capture system and EMG before and after applying tDCS in each session. All assessment will be conducted by the principal researcher, who was trained for conducting all the assessments. The overall study design is illustrated in Fig. 1. A Standard Protocol Items: Recommendations for Interventional Trials (SPIRIT) schedule is presented in Fig. 2, and a SPIRIT checklist is available in Additional file 1. The steering committee is the supervisory panel for the principal researcher, and data monitoring is done by the Research Method Advisory Group in the Institute of Health and Biomedical Innovation (IHBI) at Queensland University of Technology (QUT). The trial will be monitored on a weekly basis by the supervisory panel. The Institutional Human Research Ethics Committee approved the research. The ethics committee will be informed of any trial modifications, and these updates will be made to the trial information on the Australian New Zealand Clinical Trials Registry website. The study will be undertaken and reported based on the Consolidated Standards of Reporting Trials (CONSORT) statement for non-pharmacological treatment.

### Participants

Eighteen participants diagnosed with idiopathic PD aged 40–80 years old will be recruited from (1) Parkinson’s Queensland Incorporated (PQI), i.e. a community-based PD advocacy and support group; (2) local neurology clinics; (3) PD support groups; (4) advertisement via university website, emails and Facebook. Potential participants will be sent an information sheet explaining the details of the study and an invitation to contact one of the research team members if they wish to participate in the study.

Interested participants will initially be screened for the basic exclusion criteria, contraindications and special considerations before tDCS via a telephone screening procedure. Exclusion criteria will compromise the presence of the following: uncorrected vision impairment, heart failure, uncontrolled blood pressure, cardiovascular disease, asthma, vertigo, frequent falls, dizziness, musculoskeletal injuries within the last 18 months and orthopedic surgery within the last 12 months. The tDCS exclusion criteria include having a previous adverse reaction to transcranial magnetic stimulation (TMS)/tDCS, seizure, unexplained loss of consciousness, stroke, serious head injury, surgery to the head, any brain-related neurological illness other than PD, any illness that may have caused brain injury, frequent or severe headache, having any metal in the head, having any implanted medical device, being pregnant and having any epileptic family members. Participants meeting basic inclusion criteria for participation in the study will be scheduled to visit the movement assessment laboratory, provided with full participant information by the principal researcher on the nature of the study and asked to provide written informed consent. They will undergo a clinical eligibility screening as well as baseline clinical assessments at the IHBI, QUT. For testing at the IHBI, emergency procedures will be followed as per QUT policy. QUT provides for limited counselling services (face-to-face only) for research participants of QUT projects who may experience discomfort or distress as a result of their participation in the research. Once recruited into the study, participants will be sent reminders via emails and text messages to ensure adherence to attending the three testing sessions. Participants will be asked not to start a new intervention until they complete the trial. However, if they wish to, they will be permitted to do so, and any new intervention will be documented.

### Clinical eligibility screening

Clinical assessments will test (1) cognitive function using Addenbrooke’s Cognitive Examination Revised (ACE-R), whereby a score < 82 indicates likely dementia [40]; (2) disease severity using the Movement Disorder Society Unified Parkinson’s Disease Rating Scale (MDS-UPDRS), where the total UPDRS score is determined as the sum of the scores of all items. Each item can be rated with scores of 0 = normal to 4 = severe; the higher the total score, the more severe the disease [41].

### Clinical baseline assessment

Eligible participants will be asked to perform further clinical assessments, including:

1. Visual function including contrast sensitivity and visual acuity. Contrast sensitivity will be assessed using the Pelli-Robson chart with normal values of 1.80–2.10 at both 1 and 3 m distance for persons 40–59 years old, and 1.65–1.95 at 1 m and 1.65–2.10 at 3 m for those ≥60 years old [42] ; and the Melbourne Edge Test, where abnormal values differ for different age groups ranging from < 22 in individuals younger than 50 years to < 14 in individuals older than 80 years old [43]. Visual acuity will be assessed using the Bailey-Lovie chart set, where the values of the logarithm of the minimum angle of resolution (logMAR) range from normal (− 0.3) to poor (1.00) [44, 45].
2. Lower limb peripheral sensation (internal and external malleolus, distal phalanx of third toe, arch of foot, heel and dorsum of proximal phalanx of toe) will be assessed using a Semmes-Weinstein-type pressure aesthesiometer; the lower tactile threshold score, the better [46].
3. Mobility will be assessed using the Tinetti balance and gait test, whereby a value ≤18 suggests high risk for falls and ≥ 24 suggests low risk for falls [47].

Following the clinical assessments, questionnaires will be provided to the participants to be completed at their convenience at home, including demographic information, the International Physical Activity Questionnaire (IPAQ) [48], the Activities-specific Balance Confidence (ABC) Scale [49], a medication diary to calculate levodopa equivalent daily dose, quality of life questionnaires (Parkinson’s Disease Questionnaire (PDQ-39) [50], Parkinson’s diseases gait and falls questionnaire [51] and the UPDRS (Part 2: activities of daily living; patient questionnaire section) [52]. All questionnaires have good psychometric properties and have been used in previous PD trials [3].The participants will be asked to attend the gait laboratory for three visits with at least a 1-week interval between them and to return the completed questionnaires on the first visit. The assessments and the time points and all outcome measures at which the assessments will be taken are presented in Table 1. All questionnares and forms will be found in the online thesis, which will be available via the QUT library.

**Table 1:** Baseline and follow-up outcome measures

Outcome measure	Instrument	To be completed at convenient time	Visit 1		Visits 2–4
			Screening	Clinical	
Clinical/screening measures					
Cognitive function	Addenbrooke’s Cognitive Examination (ACE-R)		✓		
Disease severity	Unified Parkinson’s Disease Rating Scale (UPDRS) and Hoehn and Yahr scale		✓		
Visual function					
Visual acuity	Binocular visual acuity: Bailey-Lovie			✓	
Contrast sensitivity	Pelli-Robson contrast sensitivity chart			✓	
	Melbourne Edge Test			✓	
Touch sensitivity	Semmes-Weinstein monofilament test			✓	
Clinical balance and gait	Tinetti Gait and Balance Instrument				✓
Mobility	Timed Up and Go (TUG) test				✓
Gait characteristics					
Spatiotemporal parameters	Nexus 2.6; Vicon, Oxford, UK				
Gait speed (m/s)					✓
Cadence (step/min)					✓
Spatial parameters (cm)	Nexus 2.6; Vicon, Oxford, UK				
Stride length					✓
Step length					✓
Step width					✓
Maximum toe clearance					✓
Temporal parameters (s and % GC)	Nexus 2.6; Vicon, Oxford, UK				
Stride/stance/swing/single support/double support/GC time					✓
Segmental linear motion (cm)	Nexus 2.6; Vicon, Oxford, UK				
Centre of mass (COM) VT/ML					✓
Head displacement, VT/ML					✓
Pelvis displacement, VT/ML					✓
Arm swing excursion					✓
Joint kinematics (deg)	Nexus 2.6; Vicon, Oxford, UK				
Trunk flexion angle					✓
Hip flexion/extension range					✓
Knee flexion/extension range					✓
Ankle dorsiflexion/plantar flexion range					✓
Joint angles (hip, knee, ankle) of stance and swing leg at maximum toe clearance					✓
Muscles activity (per muscle)	ZeroWire, Aurion Srl, Milan, Italy				
EMG onset and offset time					✓
Duration of muscle activity					✓
Total burst activation (integral of rectified EMG)					✓
Time of peak muscle activation					✓
Questionnaires					
Balance confidence	Activities-specific Balance Confidence (ABC) Scale	✓			
Freezing of gait (FOG) and falls	PD gait and falls questionnaire (FOG section)	✓			
Physical activity	International Physical Activity Questionnaire (IPAQ)	✓			
Quality of life	The 39-item Parkinson’s Disease Questionnaire (PDQ-39)	✓			

*AP* anteroposterior, *EMG* electromyography, *GC* gait cycle, *ML* mediolateral, *VT* vertical

### Primary and secondary outcomes

The primary outcome will be gait speed, and secondary outcomes include other gait kinematics, muscle activation and functional mobility (Table 1).

#### Pre- and post-tDCS assessments

##### Three-dimensional gait assessment

Objective gait evaluation will be performed using a typical 3D assessment relying on motion capture [53, 54]. Gait speed as the primary outcome and all kinematic data will be collected using the Vicon system (Nexus 2.6; Vicon, Oxford, UK) including 12 cameras recording markers at 200 Hz within a calibration volume (e.g. L:9.3 × W:2.6 × H:2.5 m).

A total of 39 spherical markers (14 mm) will be positioned over body landmarks, based on a modified Helen Hayes marker set [3], including the head (forehead and back of the head); trunk (C7 spinus process, tenth thoracic vertebra, right scapula, jugular joint and xiphoid process); hips (anterior superior Iliac spine and posterior superior iliac spine); upper limbs (lateral border of acromion, upper arm, olecranon process of the humerus, lower arm, radial and ulnar styloids and second metacarpal head); and lower limbs (thigh, tibia, knee, ankle, second metatarsal head). Markers will be attached directly onto the skin using double-sided and hypafix tape. Marker placement will be performed by the same researcher in each session to minimize inter-tester variability. The 3D position of the markers will be expressed in the Global Coordinate System (GCS) placed approximately 1.3 m on the right side and 4.6 m behind the centre of the 10-m straight walkway.

First, the participant will be asked to walk naturally 10 times over the walkway to establish his/her self-selected walking speed. This speed will be used to set the treadmill speed during the tDCS sessions. Next, the participant’s anthropometrics will be measured and entered into the Vicon Nexus software, including body mass, height, leg length, knee and ankle width, elbow and wrist width and hand thickness.

Then, an initial recording of the participant standing still in the middle of the capture volume will be conducted for static calibration to facilitate automated detection and tracking of markers. Finally, the participant will be asked to walk six times in a straight line, over even and uneven surfaces on the walkway located in the middle of the calibration volume. Sufficient rest will be allowed in between trials to avoid participant’s fatigue.

##### Timed Up and Go (TUG) test

Basic functional mobility will be assessed with the TUG test, providing the time required to rise from a chair, walk 3 m at a comfortable pace, turn, return to the chair and sit down [55]. In the present study, participants will complete a practice trial first. Two trials will then be completed and recorded; the second trial will include a dual task [26]. The participants will be asked to count backwards in threes from a randomly chosen number between 60 and 100 while performing the TUG test.

##### Electromyography

Muscle activation will be recorded at 1 kHz using the surface EMG bilateral ZeroWire system (ZeroWire, Aurion Srl, Milan, Italy). Recordings will be taken from the right and left lower limb muscles involved in gait: tibialis anterior, soleus, lateral and medial head of gastrocnemius, rectus femoris, biceps femoris, semitendinosus and vastus lateralis. Prior to applying the surface electrodes on the belly of the muscles of interest, participants with excessive hair over the muscles of interest will be shaved, using single-use disposal safety razors. Then, the skin will be cleaned thoroughly with a cotton ball and alcohol. This reduces impedance at the electrode-skin interface and improves the clarity of the myoelectric signal. After the skin is prepared, two Ambu surface electrodes (size 8 × 22/30 × 22; electrode diameter 30 × 20; inter-electrode distance 25 mm) will be placed on the muscles of interest according to the European recommendations for surface electromyography, Surface EMG for the Non-Invasive Assessment of Muscles (SENIAM) [56]. Muscle palpation will also be done to ensure validity of electrodes placement due to the anatomical variations between individuals.

### Intervention (a-tDCS and sham tDCS) during treadmill walking

The tDCS will be delivered using a portable battery-driven NeuroConn DC Plus stimulator. To ensure that the DC stimulator is not dropped, it is placed on the treadmill panel in front of the participant. Each participant will undergo two active a-tDCS sessions and one sham tDCS session with at least 1 week interval so that we avoid carry-over effects. The washout period in studies into PD and tDCS is varied between 48 h [57] to 1 week. The results of the statistical analysis for testing carry-over effects did not show any of these effects [57]. Therefore, a 1-week interval will be an appropriate washout period.

The anode will be placed centrally over the motor strip to cover a region 10–20% anterior to Cz during treadmill walking in all three sessions. To manage the risk of falls, participants will wear a harness if required. The position and size of the anode proposed in this study have been used by Kaski et al. [30, 33, 38] in a series of studies with positive effects on gait speed [30, 33, 38]. The cathode (either 10 × 10 or 4 × 4 cm^2^) will be placed over the cerebellum, 2 cm below the inion on the median line, over the three sessions. The cathode placement in the two active tDCS sessions will be counterbalanced across the participants. Thus, one third of the participants will receive active tDCS with a large cathode first, and another one third of the participants will receive active tDCS with a small cathode electrode first; the final third of the participants will receive sham tDCS with either a small or large cathode electrode (50% each). Electrodes will be inserted in saline-soaked sponge pockets. A cap and a strap will be used to attach the electrodes over the target locations. The current will be ramped up to 1 mA over 10 s in the active stimulation conditions and held constant for 20 min before ramping down over 10 s at the end of the stimulation [18]. In the sham condition, the current will be ramped up and down, at the beginning and at the end of the 20-min tDCS session (i.e. no active stimulation is administered in between). During both stimulation conditions (active and sham) all participants are asked to walk on a level Nautilus treadmill, and they will be allowed to use the hand rails. The principal researcher will apply the tDCS electrodes and oversee all aspects of the experiment. The tDCS will be terminated immediately if the participants become uncomfortable with it. The severity of adverse effects will be assessed using a scale suggested by Brunoni et al. [58] after each tDCS session.

#### Blinding and randomization

Blinding of the principal researcher will be achieved by using the “study mode” of the DC stimulator. The study mode activates active and sham stimulation with digital codes. To minimize biasing the outcomes, an independent researcher creates a list of participants using block randomization. A randomization sequence is produced through random permutations generated by a stochastic process program in R software version 3.3.4. The size of the blocks will be set to 3 with a ratio of 1:1 so that we need six blocks of the collection (1. ABC, 2. BAC, 3. BCA, 4. CBA, 5. CAB, 6. ACB). We will draw six numbers randomly and sequentially from this collection.

In the randomization list the preprogrammed digital codes of tDCS and the size of the cathode electrode will be assigned to each participant for each session and submitted to the principal researcher via a sealed envelope. The independent researcher provides the digital code and cathode electrode size for the principal researcher prior to each tDCS session. The DC stimulator display continues to indicate the impedance and the time in the same fashion for both active and sham tDCS. The principal investigator will be blinded to the participant and experimental condition during the processing and analysing of the gait data. However, in the case of an adverse event, where it is necessary for the principal researcher to know which stimulation the participant is receiving, the principal researcher will be unblinded. Data from the identifiers will be removed and replaced by codes. However, the data will be re-identifiable by using the code or linking the participant’s details to their allocation and to their different datasets. Reliability of blinding will also be assessed after each tDCS session by asking participants about the type of stimulation they believed they had received [58]. The principal investigator will also guess and record which stimulation type the participant had received.

#### Modelling electric field intensity (EFI)

**Fig. 3:**
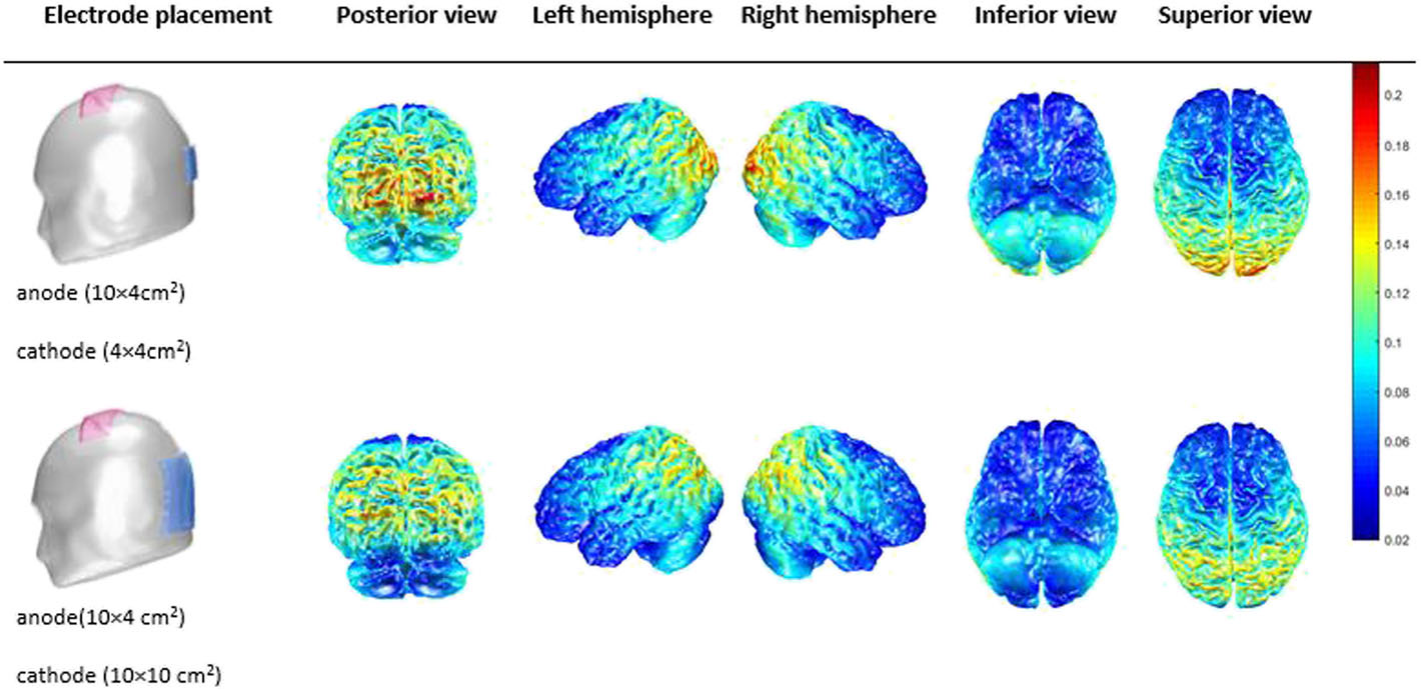
Current modelling results of electric field intensity

We modelled the EFI using a computational model generated by a MATLAB toolbox, COMETS2, to simulate the two montages. Figure 3 compares the estimated EFI generated by the large anode and either a small or large cathode [59]. This illustrates that cerebellar activation is reduced using the 10 cm × 10 cm cathode.

### Sample size

In preparation for the development of the protocol, the required sample sizes were calculated using Power Analysis and G* Power version 3.0. based on the primary outcome of gait velocity (measured by a 10-m timed walk, Kaski et al. [30]) and considering previously published cut-points for clinically meaningful difference in gait speed in people with PD: 0.02, 0.06 and 0.1 for small, moderate and large changes respectively [60]. The effect size derived from their study reflects the difference between a-tDCS and sham tDCS combined with physical training on gait velocity. To detect the difference followed by Kaski and considering a significance level of 5%, statistical power at 80%, significance level of 0.05 and delta of 0.05 and allowing for 20% drop-out across the trial, the sample size was calculated to be 18 participants, which allows for a complete cross-over randomization. We will employ some strategies suggested in the study of Little et al. (2012) for limiting data missing during conducting the trial [61].

### Gait data processing

The effects of the intervention on gait will be evaluated using a total of 21 gait parameters as listed in Table 1 [3]. The raw 3D position of each marker in GCS will be processed with the Vicon Nexus software to perform basic data processing (e.g. filtering at range of cut-off frequency of 5 Hz, detection and selection of gait events) and extraction of gait characteristics (e.g. spatial and temporal) as well as linear and angular kinematic analyses (e.g. position and orientation of segments and the centre of mass). The first and last gait cycles will be discarded to avoid considering gait initiation and termination, leaving at least three complete gait cycles for each limb available for analysis.

The gait events, including heel-contact and toe-off, will be detected manually based on the vertical displacements of the calcaneus and second metatarsal head markers respectively. All spatiotemporal parameters will be calculated using conventional methods as described by Cole [3]. Maximum toe clearance will be defined as the highest vertical displacement of the toe relative to the ground during the swing phase [62].

Sagittal plane angular kinematics of the trunk, hip, knee and ankle joints will be assessed. Trunk angle is the angle between the vector joining the sacral and C7 markers and the vertical axis of the GCS. Hip angle is defined as the angle between the vector joining the hip and knee joints and the knee and ankle joints. Ankle angle is the angle between the vector joining the knee joint and the 2nd metatarsal joint, where zero degrees is the point at which the two vectors are in a vertical position [3]. To measure segmental control, the mediolateral and vertical displacement of the head and pelvis will be assessed. Arm swing excursion will be reported by displacement of the wrists in the sagittal and frontal planes. All data in each gait cycle will be time-rescaled from 0 to 100 to facilitate averaging of all trials and reporting of events as a percentage of gait cycle. The gait data of each participant will be processed with the Vicon Nexus software, and all participants’ gait data will be merged using a code written in MATLAB (R2017a; The Mathworks, Natick, MA, USA).

### EMG data processing

All EMG data will be sampled at 1000 Hz and be filtered post-processing using a Butterworth fourth-order band pass filter (10–500 Hz). The EMG will be full wave rectified and normalized to the peak EMG recorded for each muscle. The EMG will then be aligned with the selected gait cycles and temporally normalized to 100% of the cycle. EMG will then be averaged across all gait cycles for the left and right sides. Such methods will reduce the subject-specific and situation-specific conditions that may result in signal variance. The activation profiles of each of the muscles will be assessed, including EMG onset and offset time; duration and timing of muscle activity; average time to peak muscle activation; and total burst activation (integral of rectified EMG).

The gait and EMG data processing will be conducted by the principal researcher, and all of the research team will have access to the final trial dataset.

### Statistical analysis

Sample characteristics will be summarized into the number of non-missing data, mean, standard deviation, minimum, maximum and 95% confidence interval, for quantitative variables. Number of non-missing data, frequency and proportion will be used for the description of categorical variables.

Gait speed will be the primary outcome in this study. The possible effects of participants’ physical activity level at baseline on gait speed will be investigated by performing an analysis of the Pearson correlation between gait speed (estimated using the Vicon system before applying tDCS) and physical activity level.

We will examine the within-visit effects of each tDCS on gait speed as the primary outcome and the gait kinematics and TUG time as secondary outcomes using a paired *t* test to compare pre- and post-tDSC measures.

The main effect of the three tDCS conditions (monopolar bilateral tDCS, bipolar bilateral tDCS and sham tDCS) on gait speed and gait kinematics will be examined through a linear mixed model (LMM) with random intercepts. The LMM model will contain participants nested in sequence as a random effect. In order to investigate the carry-over effect, we will compare three measures before the three tDCS procedures using one-way analysis of variance (ANOVA). When appropriate, post hoc comparisons will be carried out using a Tukey correction for multiple comparisons. The LMM will also be conducted to examine the potential interaction of time (levels: pre- and post-tDCS) and tDCS (levels: monopolar bilateral tDCS, bipolar bilateral tDCS and sham tDCS) in the two conditions of even and uneven surfaces. *P* values less than 0.05 will be considered significant, and for valid interpretation of the results the confidence interval will be reported as an indication of effect size [63]. All analysis will be performed using SPSS software (version 23; IBM Corporation, Armonk, NY, USA). Missing data will be handled using a simple imputation method [58]. This Statistical Analysis Plan (SAP) will be provided before opening the database.

## Data management

All data will be de-identified and coded. The electronic data will be stored on a secure university server which is regularly backed up and is password protected. The location on this server containing the computer files is password protected and is to be accessed only by research team members. All paper copies will be removed after a data collection session and stored in a locked cabinet in a secure, key-access laboratory at IHBI at QUT. The members of the research team, in particular those who are involved in data collection, will have the keys to access both the laboratory and the cabinets. According to the Australian Code for Responsible Conduct of Research (National Health and Medical Research Council (NHMRC), Australian Research Council (ARC), Universities Australia (UA), 2007), re-identifiable data will be stored for a minimum of 15 years after completion of the project. All digital research data will be held in a secure server at the IHBI with access only by investigators involved in the research based on QUT record management policy and the university‘s information privacy policy.

## Discussion

During the last few years, the treatment of PD has shifted from a focus on pharmacological intervention towards investigation of different non-pharmacological approaches such as tDCS. Such approaches have been motivated due to the side effects of PD medications which have included dyskinesia [28, 64, 65] and freezing of gait [2]. tDCS has been suggested as an alternative and promising adjunct treatment for PD. However, results of previous clinical trials in improving gait in PD have been equivocal, and an optimum tDCS montage has not been established. Therefore, we will systematically investigate the effects of two different montages administered during treadmill walking and their effects on gait and EMG parameters. To do so, we will use a modified montage used by Kaski [30, 33, 35, 38] to determine whether the synergetic effects of a-tDCS over both the premotor and primary motor cortices and c-tDCS over the cerebellum are more beneficial compared to the effects of a-tDCS over the premotor and primary motor cortices in PD. Furthermore, the comparison of the effects of the two montages on behavioural changes will provide information for further establishing the exact role of a-tDCS with a large electrode (monopolar set up) and a-tDCS with small cathode electrode (bipolar set-up) on gait in PD. Indeed, our initial evaluation of EFI modelling (Fig. 3) shows that the small cathode electrode over the cerebellum results in more pronounced current flow to the cerebellum, affecting its posterior, inferior and lateral aspects and the vermis of the cerebellum, while the EFI over the cerebellum with the large cathode electrode is on the lateral edges of the cerebellum.

One of the possible methodological limitations of this study will be the potential of carry-over effects between testing sessions. Consequently, we will compare the pre-test results of all conditions to ensure they are not significantly different due to possible carry-over effects. Also, we will use a self-selected most comfortable walking speed on the treadmill, which varies in each participant and might result in different activation of M1. Furthermore, we will only assess the acute effects of tDCS on the outcome measures, as long-term effects of a-tDCS on gait abilities were deemed beyond the scope of this study. Nonetheless, this study will inform future studies on the effects of optimal montage of tDCS combined with treadmill walking. If our proposed montage is effective in improving gait in PD, further research needs to be conducted to identify the long-term effects of multiple sessions of tDCS with the same parameters and concurrent accumulation effects of tDCS with other physical tasks. This may offer a significant option for the treatment of PD.

## Trial status

This trial is an ongoing project started in 2017 and is expected to be completed in November 2018.

## Additional file

Additional file 1: SPIRIT checklist. (DOCX 56 kb)

## Abbreviations

ABC: Activities-specific Balance Confidence (Scale)
ACE: Addenbrooke’s Cognitive Examination
a-tDCS: Anodal tDCS
EMG: Electromyography
GC: Gait cycle
GCS: Global Coordinate System
IPAQ: International Physical Activity Questionnaire
LMM: Linear mixed model
M1: Primary motor cortex
PD: Parkinson’s disease
PDQ-39: Parkinson’s Disease Questionnaire
PQI: Parkinson’s Queensland Incorporated
QUT: Queensland University of Technology
tDCS: Transcranial direct current stimulation
TMS: Transcranial magnetic stimulation
TUG: Timed Up and Go (test)
UPDRS: Unified Parkinson’s Disease Rating Scale
